# Two-Part Discourse Markers in Chinese: The Processing of Causal and Concessive Relations

**DOI:** 10.1007/s10936-026-10269-2

**Published:** 2026-08-27

**Authors:** Merel C. J. Scholman, Jingqi Yan, Frances Yung, Vera Demberg

**Affiliations:** 1 Institute for Language Sciences, Utrecht University, Utrecht, the Netherlands; 2 School of Foreign Languages, Zhejiang Gongshang University, Hangzhou, China; 3 Language Science & Technology, Saarland University, Saarbruecken, Germany; 4 Computer Science Department, Saarland University, Saarbruecken, Germany

**Keywords:** Discourse processing, Chinese, Connectives, Reading time, Causality, Concession

## Abstract

Prior work has shown that comprehenders can predict upcoming discourse relations based on two-part markers such as *on the one hand…on the other hand* or *true/sure…but* in English, Dutch and German. Chinese has a larger inventory of such two-part markers, and these pairs thus might have larger effects on processing. In the present study, we investigated whether Chinese readers show a similar processing advantage for two-part connectives, and whether this facilitation differs across relation types. We compare processing of causal relations to concessive relations, which are cognitively more complex. Specifically, we conducted two self-paced reading experiments testing whether concessive two-part connectives yield a greater processing advantage than causal connectives. Across both experiments, we found no evidence that the presence of the first marker facilitated processing of the second marker, nor that concessive relations elicited greater facilitation than causal relations. Bayes factor analyses provided evidence against the hypothesized interaction. These findings suggest that, unlike previous findings in Indo-European languages, two-part connectives in Chinese may not consistently enhance processing, which might be due to the greater reliance on contextual and pragmatic cues in discourse comprehension. Our results contribute to the growing body of cross-linguistic research indicating that the facilitative role of connectives is language- and context-dependent.

## Introduction

Successful language comprehension requires the reader to establish coherent links across clauses and sentences (Sanders et al., 1992). Discourse connectives play an important role in this process by signaling the relations that hold between propositions, thereby guiding readers and listeners in constructing a coherent mental representation of the text (Britton, 1994; Gernsbacher, 1997). A substantial body of research has shown that connectives can reduce processing difficulty by making relational information more explicit (e.g.,Canestrelli et al., 2013; Cozijn et al., 2011; Köhne-Fuetterer et al., 2021; Sanders & Noordman, 2000; Van Silfhout et al., 2014; Wei et al., 2019; Xiang & Kuperberg, 2015). The mechanisms underlying this facilitation remain debated: connectives may help by allowing readers to anticipate upcoming content, by making integration easier once the new information appears, or by some combination of both.

Two-part connectives, such as *on the one hand...on the other hand* in English, are of particular interest in this regard. These constructions consist of two coordinated markers whose meaning contributions are distributed across the discourse relation by marking both relational arguments explicitly. Because the first element of a two-part connective introduces the relation early, it can reduce processing effort when the second marker is encountered. Facilitation from two-part connectives may operate at a lexical level: frequent co-occurrence

patterns between the two markers may allow readers to pre-activate a specific upcoming connective form based on the first marker, thereby leading to faster processing when the second marker is encountered. The facilitation may also operate at a discourse-integration level: connectives may facilitate the construction and confirmation of coherence relations between discourse segments, thereby leading to faster processing once the relation type becomes clear to the reader (i.e., at the point of the second marker).

Previous work in English, Dutch and German has indeed found that the initial marker in such constructions can reduce processing times for the subsequent marker, suggesting a facilitative role of two-part connectives (Scholman et al., 2017, 2024, 2025; Schwab & Liu, 2020). Specifically, Scholman et al., (2017, 2024, 2025) investigated processing of contrastive two-part markers (*on the one hand…on the other hand* and their Dutch equivalents), and Schwab and Liu (2020) tested processing of concessive two-part markers (*true/sure…but* and their German equivalents). However, in English, Dutch and German, relatively few two-part marker pairs exist. This brings up the question of how generalizable the findings are to other languages, and how the relation type that the connectives mark might modulate the potential facilitative effect of the connective.

For Chinese, the role of such two-part markers remains underexplored. Chinese is interesting in this respect because it features a relatively rich inventory of two-part connectives, especially for causal and concessive relations.^1^ Chinese readers may therefore be more accustomed to encountering these forms and exploiting them more readily. In the current contribution, we investigate whether the potential facilitation observed in English extends to Chinese, and whether such effects depend on the type of relation being expressed.

So far, empirical evidence has been mixed. Xu et al. (2018) examined single concessive and causal connectives and did not find clear evidence that these Chinese connectives facilitate early comprehension, suggesting that by the time Chinese readers encounter an overt connective, they may already have inferred the relation from prior pragmatic and contextual cues. However, in a more recent study investigating single and two-part Chinese connectives, Gao et al. (2024) did find a facilitation effect of causal connectives, but found no difference in the ease of processing between sentences with two-part causal connectives and those with only the second marker. Crucially, these studies did not test whether the potential facilitation of two-part connectives might differ between causal and concessive relations, which vary in their processing complexity.

The present study addresses this gap. We investigate whether the first marker of a two-part connective facilitates processing of the second in Chinese, and whether this effect is stronger for concessive relations than for causal ones. We first present corpus data (Sect. Corpus Distributions), which provides descriptive evidence on the frequency and distribution of causal and concessive connectives in natural Chinese usage. We then report on two self-paced reading experiments (Sect. 4 and 5), designed to test our hypotheses on relation type and relational marking.

## Background

The studies we review below provide the context for asking whether two-part discourse markers can facilitate processing in Chinese. This section first discusses discourse relations and discourse processing, followed by a review of the expectations that connectives can elicit in general, and in Chinese discourse processing in particular.

### Discourse Processing and Discourse Relations

Establishing discourse relations between text segments is a key part of discourse processing. Discourse relations help comprehenders construct a mental model of how ideas and events connect. They can take many forms, including temporal, additive, contrastive, causal, and concessive links (Mann & Thompson, 1988; Sanders et al., 2021; Webber et al., 2019). The present paper focuses on the processing of causal relations and concessive relations.

In a causal relation, one clause presents the cause of an event whose outcome is described in the other (i.e., P–Q). For example, in (1), the puppy’s barking is explained as a consequence of hearing the bell.

1. The puppy heard the bell ring so she barked.

By contrast, a concessive relation sets up a causal expectation and then explicitly denies it (i.e., P→Q) (Konig & Siemund, 2000; Webber et al., 2019; Zufferey & Degand, 2024). In (2), hearing the bell would normally be expected to result in barking, yet the outcome is reversed: the puppy remains calm. In other words, the expectation raised in one clause is denied in the other (Webber et al., 2019; Zufferey & Degand, 2024).

(2) The puppy heard the bell ring. Nevertheless, she remained calm.

From a processing perspective, these two relation types pose different demands. Causal relations are generally easier to process because they align with default expectations of how events unfold in the world (Sanders & Noordman, 2000; Sanders, 2005; Zufferey & Degand, 2024). In contrast, concessive relations are typically more demanding: they require comprehenders to recognize an expected outcome and integrate an alternative outcome that violates it (Konig & Siemund, 2000; Zufferey & Degand, 2024).

This additional inferential step can result in slower reading times and greater processing costs, as documented in both offline and online measures across several languages (e.g., Köhne-Fuetterer et al., 2021; Lyu et al., 2020; Xu et al., 2018). For example, Caron et al. (1988) found that sentences containing the adversative connective *but* were remembered less accurately compared to sentences containing the causal connective *because*. Further, Köhne-Fuetterer et al. (2021) compared the processing effort for causal versus concessive relations. They found in a visual world paradigm that the presence of the connective *however* in passages such as (3) can quickly reverse comprehenders’ expectations of an expected result (getting something sweet to eat), thereby making an unexpected concession (getting something savoury to eat) expected. They also found that participants showed lower comprehension on concessive than on causal relations, suggesting that causal relations are easier to comprehend in general. In addition, concessive connectives elicited a larger P600 response than causal connectives, indicating that they require a stronger update of the discourse representation (Köhne-Fuetterer et al., 2021).

(3) Marc fancies a small snack. He feels like having something sweet. [However], he gets…

Because concessive relations are more complex than causal relations, they may offer more room for facilitation from explicit markers that help signal the relation in advance. In the current study, we investigate this by asking whether the presence of initial markers has a greater facilitative effect in concessive compared to causal relations.

### The Role of Connectives in Discourse Processing

A substantial body of psycholinguistic research has demonstrated that the presence of discourse connectives such as *because* or *however* can facilitate discourse processing (Canestrelli et al., 2013; Caron et al., 1988; Köhne-Fuetterer et al., 2021; Millis & Just, 1994; Sanders & Noordman, 2000; Traxler et al., 1997; Van Silfhout et al., 2015; Wei et al., 2019). For example, Cozijn et al. (2011) reported that explicit connectives supported readers in integrating complex discourse relations, especially when inferential demands were high. Xiang and Kuperberg (2015) found that the presence of the connective *even so* in passages like (4) can reverse comprehenders’ content expectations quickly, thereby making comprehenders more receptive to the mention of less typical responses to failing a test.

(4) Elizabeth has a history exam on Monday. She took the test and failed it. (Even so, / *\emptyset*) She went home and celebrated wildly.

However, connectives do not always yield measurable facilitation. Some studies have shown little to no effect of adding connectives, which might be explained by reader characteristics, text difficulty, relation type, connective difficulty, or whether other signals are present (e.g., Freebody & Anderson, 1983; Kleijn et al., 2019; McNamara et al., 1996; Murray, 1997). This variability suggests that the effect of connectives depends on the complexity of the relation and the availability of alternative cues. For example, corpus and experimental research has shown that non-connective signals, such as semantic verbs with implicit causal bias, negation, quantifiers, or context can guide expectations and facilitate integration (e.g., Crible, 2021; Crible & Pickering, 2020; Kehler et al., 2008; Mak & Sanders, 2013; Rohde & Horton, 2014; Scholman et al., 2020; Schwab & Liu, 2020). The presence of these alternative signals may reduce reliance on overt connectives, and could result in smaller effects in some studies compared to other studies, depending on the design of the materials.

It is also possible that the role of connectives in guiding expectations may differ across languages (Schwab & Liu, 2020; Yi & Koenig, 2021). For example, Yi and Koenig (2021) found that English and Korean speakers differ in the likelihood of producing certain causal continuations, indicating that grammatical constraints can shape discourse expectations independently of conceptual or cultural factors. Marchal et al. (2025) tested a hypothesis proposed by Blumenthal-Dramé (2021), which posits that speakers of German (a more synthetic language) rely more heavily on explicit connectives than speakers of English (a more analytic languages, as is Chinese), who are presumed to rely more on lexical information. They found no consistent evidence that German speakers showed stronger facilitation from connectives than English speakers. This goes against a cross-linguistic difference account.

The current study focuses on a particular subset of connectives: two-part discourse markers (also known as correlative conjunctions or paired connectors). The first element of such a pair can serve to establish a structural frame for what is to come, which may allow for more efficient processing once the second marker is encountered. This facilitation could arise by reducing uncertainty about the discourse relation; an effect that can be conceptualized within expectation-based accounts of language processing (e.g.,Huettig, 2015; Kuperberg & Jaeger, 2016; Levy, 2008). These accounts propose that comprehenders maintain a mental representation of the unfolding discourse and generate predictions about upcoming content based on contextual and linguistic cues. A connective may reduce uncertainty about the upcoming discourse relation or pre-activate the mental structure required to integrate the subsequent clause (see also Ferreira & Chantavarin, 2018, for a discussion of integration versus prediction). Note that expectation-based facilitation could operate either at the level of the discourse relation itself or at the level of the specific lexical marker, and the present study remains agnostic about the precise locus of the effect (but see Scholman et al., 2024, for evidence supporting the latter).

Empirical evidence from English, German and Dutch supports the hypothesis that twopart markers allow for facilitation of the subsequent relation: the presence of the first marker in a two-part construction has been found to facilitate the processing of the second marker and the relation it introduces (Scholman et al., 2017, 2024, 2025; Schwab & Liu, 2020). For example, Schwab and Liu (2020) investigated the processing of marker pairs using the English and German *true/sure…but* and *zwar…aber* constructions (see Example (5)). In a selfpaced reading paradigm, they demonstrated that processing of the connectives *but* and *aber* was facilitated when they were preceded by their lexical counterparts *true/sure* and *zwar*, respectively: reading times on the German connective *aber* and on the region following the English connective *but* were significantly faster in the paired condition compared to the single-marker condition.

(5) James likes to run outdoors. [**True**,] he has a treadmill in the living room, **but** he often jogs in parks.

Similarly, Scholman et al. (2017) examined constructions such as (6), showing that readers can generate fine-grained expectations about upcoming discourse dependencies based on the initial marker. In a story continuation study and an eye-tracking study, they found that the marker *on the one hand* (OT1H) prepared readers for a contrastive continuation, and that processing difficulty arose when the expected second contrast (*on the other hand*, OTOH) was delayed or disrupted by an intervening plausible contrast. Specifically, Scholman et al. (2017) showed that if a sentence intervened between the OT1H and OTOH segments and conveyed a plausible contrastive element (e.g., an example for (6): *But this will certainly have an effect on their friendship.*), the reading times of OTOH increased, indicating processing difficulty for encountering a subsequent contrastive segment at that point in the discourse. Hence, readers not only built, but also maintained, fine-grained predictions of upcoming contrast relations based on the first marker.

(6) Helen found some signs that Ben, her colleague and friend, has violated the company rules.

**On the one hand,** she’s thinking she should report it to a superior.

**On the other hand,** she could talk to Ben about it first.

An important consideration is what exactly the facilitative effect of two-part connectives reflects. In the present study, we operationalize facilitation as faster processing of Marker 2 when it is preceded by Marker 1. However, such an effect could arise at different levels of processing: *lexical facilitation* and *discourse-integration facilitation*. At a lexical level, Marker 1 may pre-activate a specific Marker 2 form if the two elements frequently co-occur, thereby leading to faster processing when that form is encountered. Alternatively, facilitation may arise at a discourse-integration level, where the presence of Marker 1 helps readers anticipate or construct the upcoming coherence relation, thereby easing integration once the connective or subsequent clause is encountered. Thus, facilitation could manifest either as faster processing of the connective form itself or as easier integration of the discourse relation. In practice, these processes may overlap, because the point at which Marker 2 is encountered is also the point at which the discourse relation becomes clear and can be integrated into the unfolding discourse representation. The present study therefore does not attempt to fully disentangle lexical and discourse-integration facilitation, but instead examines whether the presence of Marker 1 facilitates processing at the point where the relation becomes known. Evidence from Scholman et al. (2024) does, however, speak to these possibilities: they used the Dutch pair of discourse markers *Aan de ene kant…Aan de andere kant* (‘On the one hand…On the other hand’) and *Enerzijds…Anderzijds* (also equivalent to ‘On the one hand…On the other hand’) to test whether processing of the lexical marker for the second segment is facilitated (i) when the first segment contains a lexical marker to signal the upcoming contrast, and (ii) when that marker directly matches that of the second segment. Their findings showed a clear facilitative effect of the initial marker, while lexical repetition between the first and second markers did not yield additional facilitation. Eye-tracking data showed that the facilitative effect emerged early in processing, providing further evidence of the immediate influence that the first marker has. These results indicate that comprehenders are aware of the discourse dependency established by a discourse marker and are flexible in identifying and integrating discourse relations with different markers.

In sum, in English, German and Dutch, two-part discourse markers can significantly facilitate the processing of discourse relations. The current study extends this line of research to Chinese, focusing on whether the presence of an initial marker facilitates the processing of the subsequent marker, and whether this facilitation is modulated by the type of relation (causal vs. concessive).

### Discourse Processing and Two-Part Connectives in Chinese

Research on discourse relation processing in Chinese has highlighted differences from findings in English, German and Dutch. Xu et al. (2018) used eye-tracking and self-paced reading to investigate the processing of concessive and causal connectives in Chinese. Their results showed that concessive relations generally involve greater cognitive complexity than causal ones, and that the presence of explicit connectives does not always lead to consistent facilitation during comprehension. Specifically, connectives did not show clear early facilitation effects, but did support integration of the discourse relation. The authors suggest that discourse connectives play a significant role in establishing coherent discourse representations in Chinese, but also that different connectives have different effects on processing. Importantly, however, their study investigated single connectives, without considering whether two-part connectives might offer additional facilitation.

Chinese has a rich inventory of discourse connectives, including many two‑part connective pairings (Wan et al., 2024). One notable property of these connectives is their flexibility in form and position within a sentence. They may occur as a full pair distributed across two clauses, or individually, with only one relational clause overtly realized (Zhu, 1982). For example, the causal pair *yinwei…suoyi* (‘because…therefore’) can appear with both markers, as in (7), or with only the first or second element, as in (8) and (9) respectively.

**Table:** 

(7)	**Yinwei** Xiaoming shengbing le,	**suoyi**	ta mei qu shangxue
	because Xiaoming sick	ASPECT MARKER, therefore he not go school	
	‘Because Xiaoming was sick, therefore he did not go to school.’		
(8)	**Yinwei** Xiaoming shengbing le,		ta mei qu shangxue
	because Xiaoming sick	ASPECT MARKER, therefore he not go school	
	‘Because Xiaoming was sick, he did not go to school.’		
(9)	Xiaoming shengbing le,	**suoyi**	ta mei qu shangxue
	because Xiaoming sick	ASPECT MARKER, therefore he not go school	
	‘Xiaoming was sick, therefore he did not go to school.’		

Single occurrences of the second marker are generally more frequent than full pairs or uses of the first marker alone (Hsieh, 1999; Zhang, 2012). Some connectives appear more commonly in the first clause to signal an upcoming discourse relation, while others are strongly associated with the second clause. In addition, the strength of the bond between paired connectives varies across constructions, which in turn may influence how strongly the presence of the first element facilitates processing of the second.

In the current study, we focus on two pairs of two-part markers: *yinwei…suoyi* (‘because…therefore’) and *suiran…danshi* (‘although…but’). While both markers can occur independently, the patterns differ across relation types, as we will see in the corpus data presented in Sect. "Discourse processing and discourse relations". The first marker can often stand alone without difficulty in causal relations, as in (8). In concessive relations, however, the first marker is followed by the second marker much more frequently. This asymmetry suggests that the presence of a concessive first marker may more strongly cue the expectation of a second marker than in causal constructions, potentially leading to differential processing effects.

The only study experimentally investigating the facilitative effect of two-part connectives in Chinese considered the processing of causal two-part connectives: Gao et al. (2024) investigated whether the presence of both Marker 1 and Marker 2 of a causal relation (e.g., *yinwei…suoyi*) yielded processing advantages compared to only Marker 2 (*suoyi*) or no marker at all. Their results showed facilitation of a causal connective on early processing measures, but, crucially for the current study, their results showed no significant differences in reading times between the condition in which both Marker 1 and Marker 2 were present or the condition in which only Marker 2 was present. This could mean that the first marker may not substantially facilitate online discourse processing in Mandarin, or that there is no effect of facilitation in causal contexts specifically.

Taken together, these findings raise the possibility that the processing benefits of connectives in Chinese may be more limited or context-dependent than in English. However, the interaction between relation type (causal vs. concessive) and connective pairing (full vs. partial pairs) remains unexplored.

### Current Study

The current study examines whether the processing advantage of two-part connectives reported in English extends to Chinese, and whether such effects are modulated by the type of discourse relation that the connectives signal.

We first present a corpus study, which provides descriptive evidence on the frequency and distribution of causal and concessive connectives in natural Chinese usage and, in particular, on the strength of lexical associations between Marker 1 and Marker 2 that may support lexical-level facilitation. We then report two self-paced reading experiments designed to test these predictions in a 2 × 2 design crossing relation type (causal vs. concessive) and relational marking (two-part vs. Marker 2 only). The materials were adapted from English stimuli used in Köhne-Fuetterer et al. (2021), who compared the processing of causal and concessive connectives.

If readers activate an expectation for a discourse marker on the basis of the first element of a pair, we should observe faster reading times on the second marker when it is preceded by Marker 1 than when it appears in isolation. We therefore predict a main effect of relational marking: both causal and concessive relations should show facilitation from the presence of an initial marker. At the same time, not all discourse relations are equally easy to process (see Sect. Discourse processing and discourse relation). We therefore expect a main effect of relation type, with concessive relations eliciting longer reading times than causal relations.

Combining these predictions leads to the expectation that the processing benefit of Marker 1 may be greater in concessive than in causal relations, yielding an interaction between relation type and relational marking. Two motivations underlie this prediction. First, because concessive relations are cognitively more demanding, readers may rely more strongly on explicit connective cues. Second, as shown in our corpus study, concessive Marker 1 and Marker 2 exhibit tighter distributional coupling than their causal counterparts, increasing the likelihood that readers can anticipate the upcoming connective form. While these motivations cannot be fully disentangled in the present design, they may lead to facilitation effects at different points in the time course of processing. Distributional coupling between Marker 1 and Marker 2 would primarily predict facilitation at the second marker itself, reflecting lexical pre-activation of the expected connective form. In contrast, effects driven by the greater cognitive demand of concessive relations would be expected to emerge at the point where the relation is integrated into the discourse representation, potentially in the spillover region following the connective. Because the appearance of Marker 2 also coincides with the point at which the relation becomes explicit, these effects may overlap in the current paradigm.

## Corpus Distributions

We first examine how causal and concessive discourse relations differ in their connective pairing patterns within natural language use. The distribution patterns observed in the corpus data can provide insights into how the presence of an initial discourse marker might facilitate the processing of upcoming content across these relation types.

### Data

We analyzed the frequency and distribution of causal and concessive connectives in the Brown family Chinese corpus ToRCH2019^2^, a one-million-word Chinese corpus modeled after the English Brown Corpus. It comprises 671 texts from four major genres (press, general prose, academic, and fiction) published in 2019. We identified connectives marking causal and concessive relations based on frameworks by Xue (2005) and Zhang (2012), as listed in Table 1. We extracted all sentences containing at least one such connective, ensuring that each instance clearly expressed either a reason-result (causal) or a negative cause-result (concessive) relation. In sentences with paired connectives, Marker 1 always appeared in the first clause (indicating the cause), and Marker 2 in the second clause (indicating the consequence).

**Table 1:** Discourse connectives for causal and concessive relations in natural corpus

Relation	Marker position	Discourse connectives
Causal	Marker 1 (reason)	因为 (yinwei),由于 (youyu),因 (yin)
	Marker 2(result)	所以 (suoyi),以致 (yizhi),于是 (yushi),因而 (yiner),因此 (yinci),从而 (conger),故 (gu),遂 (sui),以至于 (yizhiyu),故而 (guer), 就 (jiu)
Concession	Marker 1	虽然 (suiran),虽说 (suishuo),虽 (sui),尽管 (jinguan)
	Marker 2-ARG2-as-denier	但是 (danshi),但 (dan),可是 (keshi),可 (ke), 却 (que),然而 (raner),不过 (buguo),而 (er),然 (ran),也 (ye)

### Results

Tables 2 and 3 report the frequencies of all Marker 1 and Marker 2 occurrences, as well as their co-occurrence patterns. The most common pairings were *yinwei…suoyi* for causal and *suiran…dan/danshi* for concessive relations. Given our focus on these specific pairs, we

**Table 2:** Frequency and percentage of marker 1 and marker 2 and their combinations in causal relations

	M2-suoyi	M2-other	No M2
M1-yinwei	50 (10.7%)	55 (11.8%)	362 (77.5%)
M1-youyu	24 (10.3%)	11 (4.7%)	197 (84.9%)
M1-yin	9 (10.5%)	2 (2.3%)	75 (87.2%)
No M1	243 (30.0%)	567 (70.0%)	–

**Table 3:** Frequency and percentage of marker 1 and marker 2 and their combinations in concessive relations

	M2-suoyi	M2-other	No M2
M1-suiran	285 (73.8%)	73 (18.9)	28 (7.3%)
M1-suishuo	9 (81.8%)	2 (18.2%)	0(0%)
M1-sui	62 (53.0%)	35 (29.9%)	20 (17.1%)
M1-jinguan	99 (69.7%)	28 (19.7%)	15 (10.6%)
No M1	1030 (61.1%)	656 (38.9%)	–

examined their frequency relative to other connective combinations. To account for common usage, we combined frequencies of *danshi* and its simplified variant *dan*.

Our analysis focused on two research questions: (i) To what extent do Marker 1 and Marker 2 tend to co-occur in each relation type? (ii) Is this co-occurrence tendency comparable across causal and concessive relations?

*Marker 2 prevalence across relations.* For both relation types, Marker 2 appears more frequently on its own than as part of a pair. That is, the majority of discourse relations are expressed without an explicit Marker 1. This suggests that readers are generally accustomed to interpreting these relations based on Marker 2 alone.

We further compared the frequency of paired versus unpaired uses of two Marker 2 connectives: *suoyi* (causal) and *danshi/dan* (concessive). A chi-squared test found no significant difference in pairing configurations across relation types (*χ*^2^ = 1.378, p = 0.24, odds ratio = 0.774, df = 1). Specifically, 25% of *suoyi* instances were paired with a Marker 1, while 74% occurred alone. Similarly, 30% of *danshi/dan* instances were paired, and 69% were unpaired. Thus, both Marker 2s show a similar tendency to appear independently, suggesting that their distributional patterns are broadly comparable in terms of unpaired use.

*Strength of pairing.* Although Marker 2 is often used alone, the strength of co-occurrence between Marker 1 and Marker 2 differs substantially by relation type. Here, individual connectives are treated as lexical realizations of broader causal and concessive relations, allowing item-level co-occurrence patterns to serve as proxies for relation-level dependency. In causal relations, the presence of Marker 1 (*yinwei*) rarely predicts the presence of Marker 2: among sentences with *yinwei*, only 10% of cases include *suoyi* as Marker 2, with 11% containing other Marker 2 types, and a substantial 77% lacking any Marker 2. This suggests that *yinwei* is generally sufficient to signal the relation on its own. In contrast, concessive relations exhibit a much stronger pairing tendency: when *suiran* appears as Marker 1, 73% of the following clauses contain *danshi/dan* as Marker 2, 18% contain other Marker 2 types, and only 7% lack a Marker 2. This strong tendency toward pairing suggests that concessive Marker 1s create a stronger expectation for an explicit continuation.

### Implications of Corpus-Based Findings

In sum, both causal and concessive coherence relations are frequently expressed using only Marker 2. However, concessive relations display a stronger interdependence between Marker 1 and Marker 2. That is, when a concessive Marker 1 appears, it is much more likely to be followed by a Marker 2 than in the causal case.

The patterns for co-occurrence of concessive markers fit the patterns for contrastive and concessive markers found in Dutch, German and English. For instance, in Dutch the presence of the first marker of the connective pairs *aan de ene kant...aan de andere kant* and *enerzijds...anderzijds* (both meaning ‘on the one hand...on the other hand’) predicts the presence of the second marker relatively reliably (73% and 88% co-occurrence, respectively, Scholman et al., 2024). In English, the pair *on the one hand...on the other hand* are similarly reliably paired in natural text (83% co-occurrence, Scholman et al., 2025). The pair *true/sure...but* and its German equivalent *zwar...aber* show comparable pairing patterns, with German *zwar* appearing as part of a concessive relation in 96% of instances, and English *true/sure* appearing as part of a concessive relation in 86% and 83% of corpus instances, respectively (Schwab & Liu, 2020). There is no data on causal two-part connective pairings in any of these languages, so we cannot determine if the trend that the causal pairing is less strong than the concessive pairing replicates cross-linguistically.

The distributional patterns identified in this corpus study primarily speak to potential lexical-level facilitation, as stronger Marker 1–Marker 2 associations increase the likelihood that readers can pre-activate a specific upcoming connective form. Specifically, the findings from our corpus study suggest that a concessive Marker 1 may more strongly constrain expectations for the explicitness of upcoming discourse structure compared to a causal Marker 1, potentially influencing real-time processing. If this is the case, we should observe an interaction effect between relation type and marking: readers encountering a concessive Marker 1 would have stronger expectations for an upcoming Marker 2 compared to when encountering a causal Marker 1, leading to faster processing times at the point of encountering Marker 2. This is tested in a self-paced reading study.

## Experiment 1: Self-Paced Reading Study

### Participants

Eighty native speakers of Chinese (age range 19–49; mean age 29.68; female 52) participated in the self-paced reading experiment. Participants were recruited via Prolific and received 3.5 GBP for the participation. All participants indicated that they were born in China and used Chinese as their first language. They reported no literacy difficulties. They were not informed the purpose of the experiment. Data from 2 participants were excluded because they scored lower than 70% accuracy on the comprehension questions.

### Materials

The experimental items consisted of 24 prompts in a 2×2 design: Marker 1 (present/absent) and relation type (causal/concessive). These four conditions are illustrated in Table 4. We opted to use a smaller number of items and present these to a larger number of participants to minimize a possible effect of expectation adaptation throughout the study (Fine et al., 2013; cf. Scholman et al., 2024, 2025).

**Table 4:** An example item demonstrating causal and concessive manipulation and region segmentation

	Causal version	Concessive version
Precontext: Arg1: Arg2: Postcontext: Translation:	刘文给自己/设计了/两种纹身图案, /一种 是/植物纹饰, /另一种是/航海纹饰。/ [因为_**Marker 1**_*\emptyset*] 他的女朋友/觉得/植物纹饰/非常特别, /且容易/绘制, / [所以**Marker 2**] 刘 文 最 后Spill1/决 定让_Spill2_/纹 身 师/纹 一 个/[枫 叶 图 案]/来彰显/他的个性。/ 刘文/对这个纹身/很满意。 Liu Wen himself/ designed / two tattoo patterns,/ one was/ plant patterns,/ the other was/ nautical patterns. /[**because/***\emptyset*] His girlfriend/ thought/ plant patterns / were very special/ and were also easy/ to draw,/ [**so**]/ Liu Wen finally/ decided to let/ the tattoo artist /tattoo a /maple leaf pattern /to show/ his personality. /Liu Wen was very satisfied/ with this tattoo.	刘文给自己/设计了/两种纹身图案, /一种 是/植物纹饰, /另一种是/航海纹饰。/ [虽然_**Marker 1**_*\emptyset*] 他的女朋友/觉得/植物纹饰/非常特别, /且容易/绘制, [但是**Marker 2**] 刘 文 最 后Spill1/决 定让_Spill2_/纹 身 师/纹 一 个 [帆 船 图 案]/来彰显/他的个性。/ 刘文/对这个纹身/很满意。 Liu Wen himself/ designed/ two tattoo patterns,/ one was/ plant patterns,/ the other was/ nautical patterns. /[**although/***\emptyset*] His girlfriend/ thought/ plant patterns/ were very special/ and were also easy/ to draw,/ [**but**]/ Liu Wen finally/ decided to let/ the tattoo artist/ tattoo a/ boat pattern/ to show/ his personality./ Liu Wen was very sat- isfied/ with this tattoo.

Each item consisted of a pre-context sentence that introduced two alternatives (e.g., “plant patterns” vs. “nautical patterns”). This was followed by a second sentence, with a connective indicating either a causal or concessive relation, and the content disambiguating between the two options introduced in the pre-context. Finally, a post-context sentence concluded the discourse.

The materials were modified translations from prompts used by Köhne-Fuetterer et al. (2021). Several adaptions were made to serve the aims of the current study. First, to create the condition involving the presence of Marker 1, either connective Marker 1 (因为 “because” or 虽然 “although”) or no Marker 1 preceded the first relational argument. The corresponding Marker 2 (所以 "so" “vs 但是 “but”) was consistently included as the target marker at the beginning of the second relational argument. Second, because explicit two-part markers are pragmatically marked in Chinese and tend to occur in discourse contexts with sufficient informational load, their use in very short stimuli can sound heavy or infelicitous. We inserted a short additive clause (e.g., with “and” or “also”) between the two relational arguments, thereby enlarging the discourse and making the presence of both Marker 1 and Marker 2 pragmatically licensed. Third, after Marker 2, the clauses always include a subject and an adverbial to ensure structure consistency across items.

We conducted a coherence rating test to select items from an original set of 44 items. We aimed to select items that were comparably and maximally coherent across all conditions. Eighty-six participants completed the rating task using a five-point Likert scale (1 for incoherent and 5 for perfectly coherent) on a Chinese online questionnaire platform. Each participant was assigned to one list of eight lists, containing 22 critical items across four conditions, as well as 12 filler items with incongruent causal and concessive relations. Participants only rated the critical items in one condition. From the collected data, 24 items were selected based on mean ratings above 2.5 and minimal differences between conditions. The mean rating score for causal relations with Marker 1 condition was 4.15 (SD = 1.15), while the score without Marker 1 was 4.14 (SD = 1.15). For concessive relations, the mean rating scores with Marker 1 condition was 3.59 (SD = 1.30), and without Marker 1 condition was 3.49 (SD = 1.30).

The final set of 24 items were divided into four lists following a Latin square design. Additionally, 72 three-sentence discourses were included as fillers, among which 24 fillers contained a range of other discourse markers and 48 fillers contained other relations but without markers. In total, 96 items were presented to each participant in a pseudo-randomized order to ensure that no two consecutive critical items or identical critical conditions appeared sequentially. Participants were randomly assigned to one of the four lists. Furthermore, half of the items (all critical items and 1/3 of fillers) were followed by a yes-or-no comprehension question.

### Procedure

Participants were recruited via Prolific and then directed to a website hosted on PCIbex (Schwarz & Zehr, 2021). There, they completed the moving-window self-paced reading experiment using their own devices and browsers. All items were displayed in a monospaced font on a white background. Each trial began with a fixation star at the center of the screen. Upon pressing the space bar, a series of underscores appeared, each corresponding to the length of the regions. As participants pressed the space bar, the new region of phrases or words was revealed, while the previous region was replaced by underscores. All the items were presented centrally on the screen. For half of the items, participants were required to answer a yes-or-no comprehension question by pressing either 'j’ for Yes and ’f’ for No on the keyboard. A short break was included midway through the experiment, during which participants could proceed by clicking a ‘‘proceed’’ button. On average, the entire experiment lasted approximately 20 min.

Table (4) demonstrates the region segmentation of the critical item used in the movingwindow self-paced reading task. Slashes indicate the boundaries between regions.The items were displayed across multiple lines on the screen. Marker 1 and Marker 2 were always presented as individual chunks. Additionally, Marker 2 was never displayed at the first or final region of a line. The region following Marker 2 always included both a subject and an adverbial to maintain structural consistency across items.

**Fig. 1:**
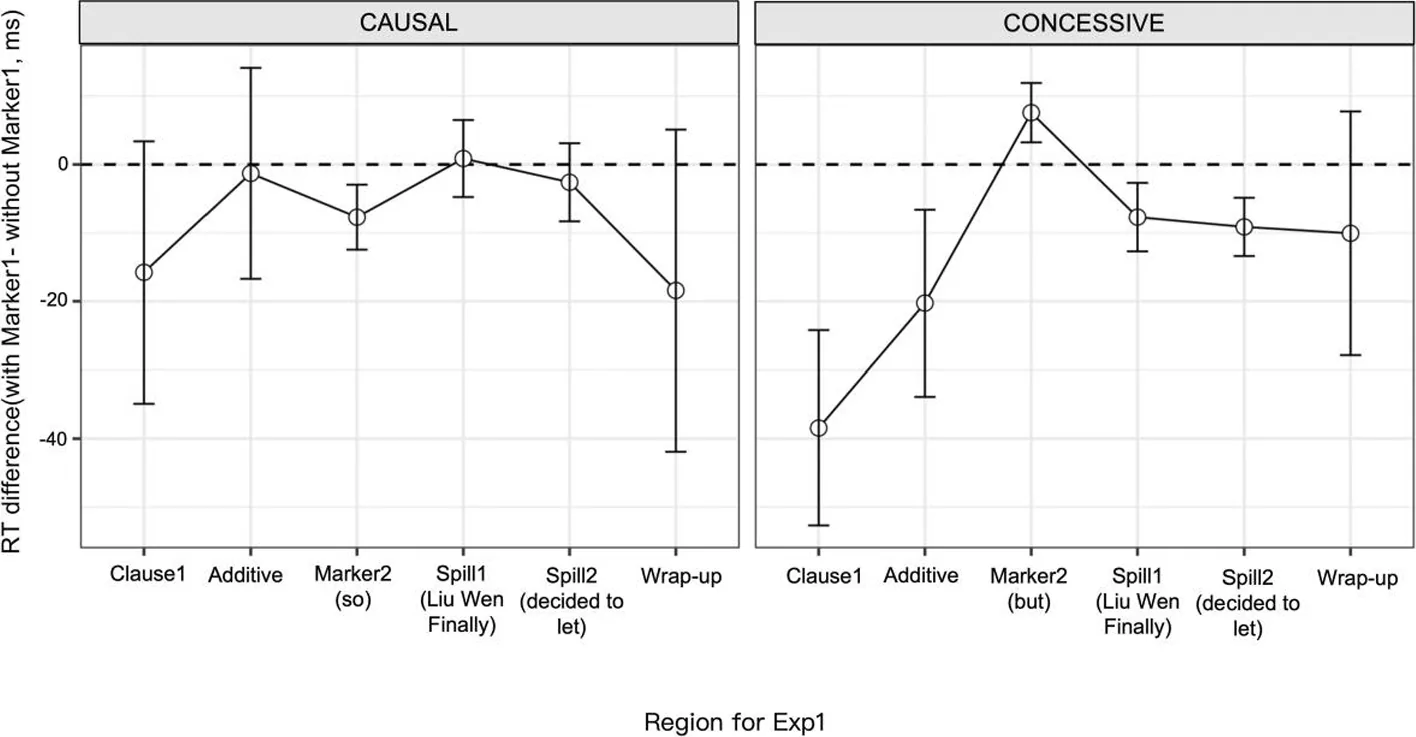
Mean difference in reading times and error bars (SE) between the condition with Marker 1 and the condition without Marker 1, Exp. 1. Negative values indicate that the region was read faster in the condition with Marker 1

The critical region is the Marker 2 region, which is the point where the reader receives the signal that the second clause of the relation will now be presented and can be integrated with the first clause. The spillover region is the primary region where the reader integrates the discourse relation signaled at Marker 2 with the prior clause. Because this integration takes time and may not occur immediately at Marker 2, this region is the best window into how expectations set up by Marker 1 influence real-time comprehension. We had pre-registered analysis on these two regions, but also present an additional, exploratory analysis of spillover region 2 (Spill2), based on the pattern visible in Fig. 1.

### Data Processing and Analysis Procedure

We first calculated each participant’s comprehension accuracy and removed participants who produced correct answers less than 70% of all trials. This resulted in the exclusion of 2 participants. For the remaining participants, the reading time data was included in the analysis. For all critical regions, RTs above 2000 ms and below 150 ms were excluded first. Next, outliers were removed if they fell beyond 2.5SD from the mean per region. The overall exclusion rate is 3.19%.

We fit Bayesian linear mixed-effects models for each region separately, using the brms package Bürkner (2017) in R. Response times (in milliseconds) were modelled using Bayesian linear regression with a lognormal likelihood. The conditions for Marker and Relation were deviation-coded (marker present, causal relation: both −0.5) and included as fixed effect along with their interaction. Participants and items were specified in the models as random effects, along with variance–covariance matrices. A centered covariate of trial order was included to account for any variance due to participants’ reading times speeding up over the course of experiment. A regularizing LKJ(2) prior was specified on the random effects correlation to ensure that extreme correlations are downweighted (Lewandowski et al., 2009).

Four chains were run using 10,000 iterations, including 2,000 warm-up, to ensure proper convergence. Model diagnostics were checked using trace plots and R-hat values, which all indicated satisfactory convergence, with R-hat values below 1.01 for all parameters. We report posterior estimates and 95% credible intervals (CRI).

The Bayes factor quantifies the support for or against a model with the effect of interest over another model without the effect of interest. Specifically, Bayes factors indicate whether the data support a genuine absence of facilitation, rather than reflecting low statistical power. Table (5) provides a guideline for interpreting the Bayes factor, following Lee and Wagenmakers (2014). To focus our Bayesian evidence quantification, we computed Bayes Factors only for effects whose 95% credible intervals excluded zero or were near exclusion, as effects including zero cannot provide conclusive evidence.

**Table 5:** Guidelines for the interpretation of the Bayes factor

Bayes factor (BF_10_)	Interpretation
> 100	Extreme evidence for M1
30–100	Very strong evidence for M1
11,232.00	Strong evidence for M1
46,298.00	Moderate evidence for M1
46,082.00	Anecdotal evidence for M1
1.00	No evidence
36,951.00	Anecdotal evidence for M0
1/10–1/3	Moderate evidence for M0
1/30–1/10	Strong evidence for M0
1/100–1/30	Very strong evidence for M0
> 1/100	Extreme evidence for M0

The order of 1 and 0 in BF_10_ indicates that we look at support for Model 1 over Model 0

An important feature of the Bayes factor is that it is sensitive to the prior distribution of the effect of interest; mildly informative priors can strongly bias the Bayes factor. Following recent recommendations for Bayesian model comparison (e.g., Mertzen et al., 2024), we therefore compared models with different priors against null models excluding the term of interest, while retaining the rest of the model structure (i.e., other main effects and interactions). Specifically, we specified three priors to reflect varying degrees of prior belief in facilitation: (1) a more informative prior *N* (0, 0.05), which indicates the evidence for or against the effect under an a priori belief that the effect size is relatively small; (2) a wider prior *N* (0, 0.1), which captures greater uncertainty about the effect size by permitting a broader range of plausible values; and (3) a directional prior *N* (0.05, 0.02), which assumes a small but positive facilitative effect specifically for the interaction between Marker 1 presence and relation type, reflecting stronger prior belief in both the direction and magnitude of this effect.

### Results

**Fig. 2:**
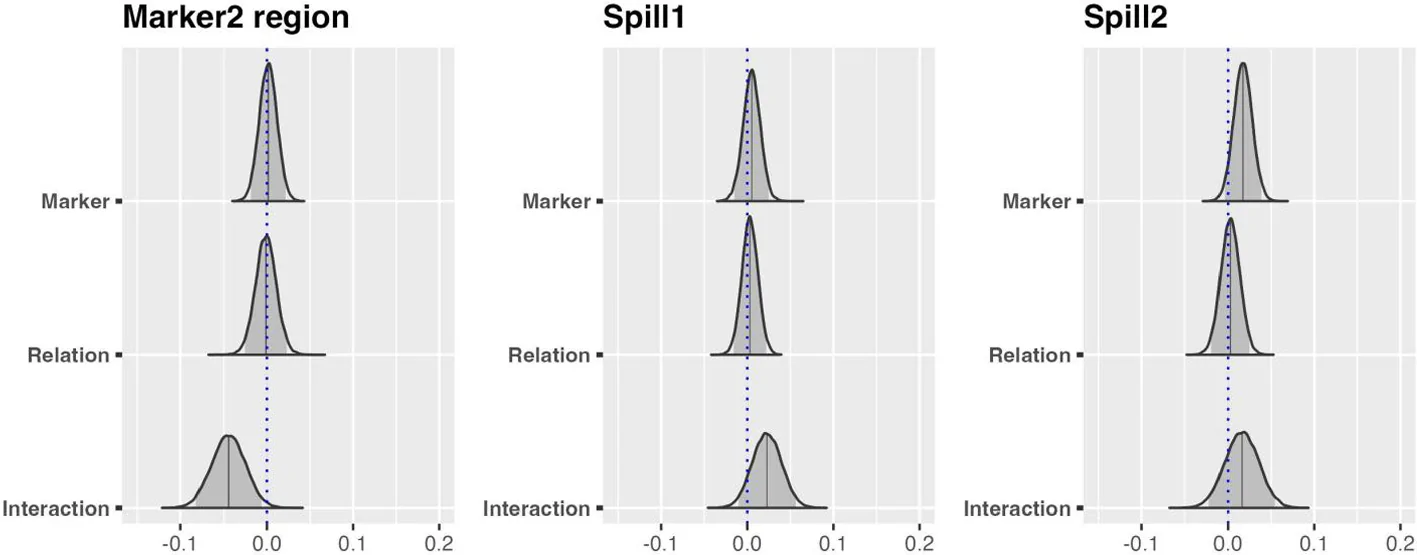
Posterior distributions for the comparisons between conditions, Exp. 1. The darker vertical line shows the median posterior effect estimate; the light gray area indicates the 95% credible interval

Figure 1 presents the difference in reading times between conditions for the first clause of the relation (excluding Marker 1), the additive clause, the Marker 2 region, the two subsequent spillover regions and the remaining part of the argument 2 across conditions. We include the summed reading times of the chunks making up the content between Marker 1 and Marker 2 (Clause1 and Additive) in Fig. 1 for completeness, following the suggestion of a reviewer. The descriptive pattern suggests that these regions were read faster when Marker 1 was present than when it was absent. However, as these regions were not part of our a priori hypotheses and were not included in the statistical analyses, we refrain from drawing strong conclusions from this pattern. We return to this in the General Discussion. Posterior distributions of the estimated parameters are shown in Fig. 2, and full model results and a raw reading time plot are included in the Appendix (Table 7 and Fig. 7).

Looking first at the connective region, the analysis indicated that there was no main effect of marker; the main effects were centered around zero. However, the model results show an interaction effect at this region ($\hat{\beta }$  = 0.04, 95% CRI = (0.00, 0.08)). As shown in Figure 2, this was a cross-over interaction: Marker 2 was read faster when Marker 1 was present in causal relations, but slower when Marker 1 was present in concessive relations. This effect is in the opposite direction of the hypothesized interaction (H3), which predicted greater facilitation from Marker 1 in concessive relations due to their higher complexity.

Figure 1 suggests a possible numerical reversal of this pattern in the spillover regions: in concessive relations, spillover region 1 and especially spillover region 2 were read slightly faster when Marker 1 was present. We therefore also ran an exploratory analysis on Spill2. However, model results for these regions indicated no meaningful effects: all main effects and interactions were centered around zero, as illustrated in Fig. 2.

**Fig. 3:**
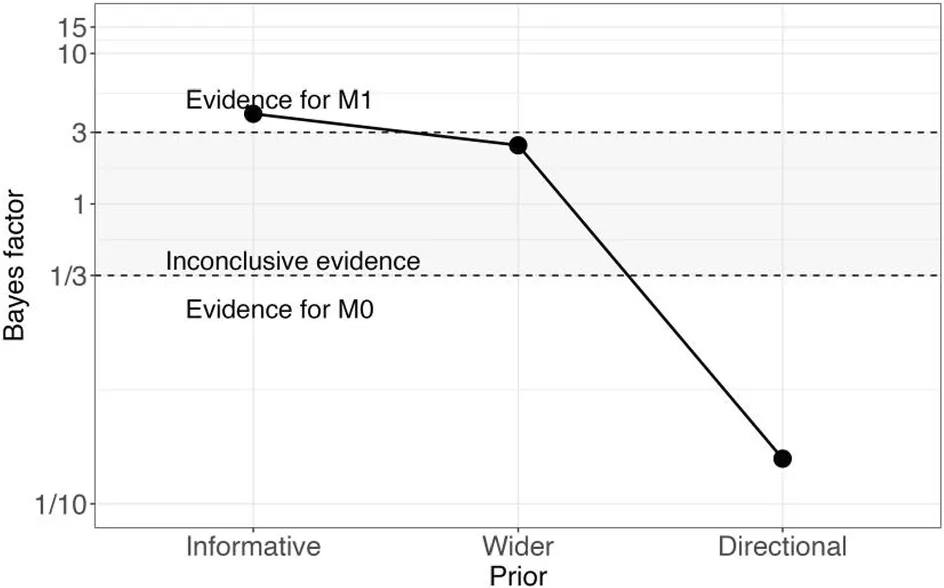
Bayes factor results for Model 1 over Model 0 for the interaction effect at the Marker 2 region, Exp. 1

*Bayes factor.* To quantify the strength of evidence for the interaction effect at the Marker 2 region, we conducted Bayes factor analyses comparing models that included the interaction term (Model 1) to those that did not (Model 0). Figure 3 shows Bayes factors (BF_10_) for three prior specifications. Results indicated anecdotal to moderate evidence in favor of an.

interaction effect, but the BF_10_ for the directional prior indicates that this effect goes in the opposite direction from our prediction. This pattern of Bayes factors suggests that the observed interaction effect contradicts the directional hypothesis and instead supports an effect in the reverse direction; namely, facilitation from Marker 1 in causal relations but increased cost in concessive ones.

### Discussion Experiment 1

The study was designed to test whether readers predict the lexical Marker 1 to Marker 2 in causal and concessive relations and whether such predictions vary to a different degree in the two relations. The results provide limited support for our hypothesis that explicit discourse markers facilitate processing. While no reliable main effects of marker presence or relation type were found, the analysis revealed a weak cross-over interaction on the Marker 2 region: causal relations were read faster when preceded by Marker 1, whereas concessive relations were read more slowly. This pattern is inconsistent with our prediction that concessive relations, due to their greater complexity, would benefit more from pre-activation via Marker 1. Note, however, that the estimated difference ( 9 ms, 2.8% of mean RT) is considerably smaller than the 50 ms effect (9% of mean RT) reported in Dutch (Scholman et al., 2024) and English (Scholman et al., 2025) for the pair *on the one hand…on the other hand* and its Dutch counterpart, indicating that if such facilitation exists in Chinese, it is likely to be very small. Moreover, a numerical reversal of this effect appeared in the spillover regions, but model-based analyses indicated no credible evidence for effects beyond Marker 2. These findings suggest that facilitation from discourse markers may be sensitive to relation type, but the effect appears to be quite small and would need further investigation.

One possible explanation for these unexpected findings is that the materials used in Experiment 1 may have introduced excessive structural complexity. Specifically, the critical items included an intervening additive clause between Marker 1 and Marker 2, which increased the linear distance between the two components of the discourse relation. This distance may have disrupted readers’ ability to maintain or retrieve the discourse expectation initiated by Marker 1, thereby limiting predictive facilitation at Marker 2. Indeed, Scholman et al. (2025) reported that including an intervening clause between *on the one hand…on the other hand* diminished the facilitative effect of Marker 1 upon encountering Marker 2. We therefore conducted a follow-up study, in which we employed a similar design to Experiment but using simplified materials: the additive clause was removed, reducing the distance between Marker 1 and Marker 2. This change aimed to reduce memory load, thereby increasing the likelihood that readers would recognize the paired structure and show the predicted facilitative processing effects. We also included more participants for increased power (120 instead of 80 participants), to make it more likely to detect smaller effects such as the crossover interaction observed in this Experiment.

## Experiment 2: Follow-Up Self-Paced Reading Study

### Participants

One hundred and twenty native speakers of Chinese (age range 20–52; mean age 29; female 77) participated in the self-paced reading experiment. Participants were recruited via Prolific and received 3.5 GBP for the participation. Data from 4 participants were excluded since they scored lower than 70% accuracy on the comprehension questions.

### Materials

The materials were adapted from those used in Exp1. The additive clause between the initial clause containing marker 1 and the subsequent clause containing marker 2 was removed for the critical items. All other elements remained unchanged. Table 6 provides an example of the items for both relations.

**Table 6:** An example item demonstrating causal and concessive discourse relations in Experiment 2

	Causal version	Concessive version
Precontext: Arg1: Arg2: Postcontext: Translation:	刘文给自己设计了两种纹身图案, 一种是植 物纹饰, 另一种是航海纹饰。 [因为/*\emptyset*] 他的女朋友觉得植物纹饰非常特别, [所以] 刘文最后决定让纹身师纹一个 [枫叶图案] 来彰显他的个性。 刘文对这个纹身很满意。 Liu Wen himself designed two tattoo patterns, one was plant patterns, the other was nautical patterns. [**because/***\emptyset*] His girlfriend thought plant patterns were very special, [**so**] Liu Wen finally decided to let the tattoo artist tattoo a maple leaf pattern to show his personality. Liu Wen was very satisfied with this tattoo.	刘文给自己设计了两种纹身图案, 一种是植 物纹饰, 另一种是航海纹饰。 [虽然/*\emptyset*] 他的女朋友觉得植物纹饰非常特别, [但是] 刘文最后决定让纹身师纹一个 [帆船图案] 来彰显他的个性。 刘文对这个纹身很满意。 Liu Wen himself designed two tattoo patterns, one was plant patterns, the other was nautical patterns. [**although/***\emptyset*] His girlfriend thought plant patterns were very special, [**but**] Liu Wen finally decided to let the tattoo artist tattoo a boat pattern to show his personality. Liu Wen was very satisfied with this tattoo.

### Data Processing and Analysis Procedure

The procedure, data processing and analysis procedure were identical to that of Experiment 1. We preregistered an analysis on the Marker 2 region as well as spillover regions 1 and 2, to account for possible effects to appear later, as suggested by the pattern visible in Fig. 1. For all critical regions, RTs above 2000 ms and below 150 ms were excluded first. Next, outliers were removed if they fell beyond 2.5SD from the mean per region. The overall exclusion rate is 4.4%.

### Results

**Fig. 4:**
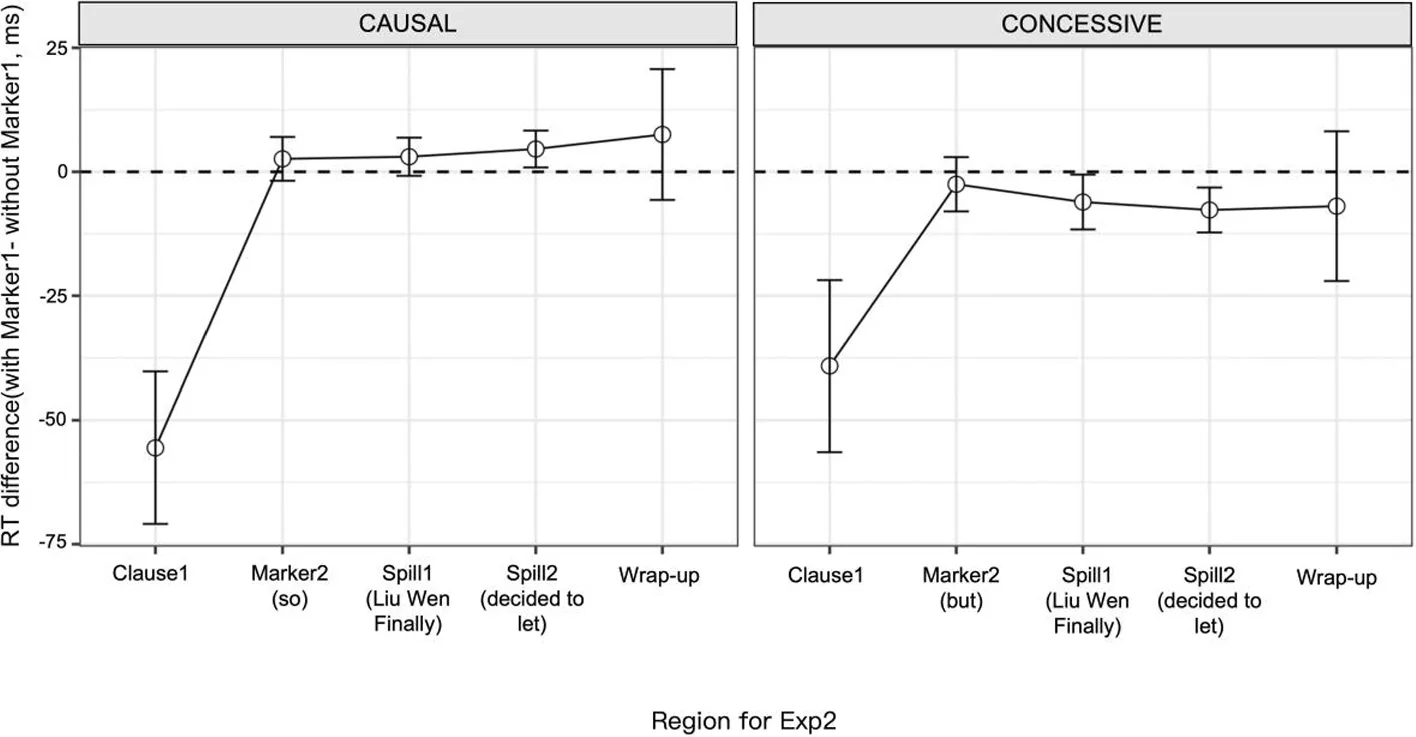
Mean difference in reading times and error bars (SE) between the condition with Marker 1 and the condition without Marker 1, Exp. 2. Negative values indicate that the region was read faster in the condition with Marker 1

**Fig. 5:**
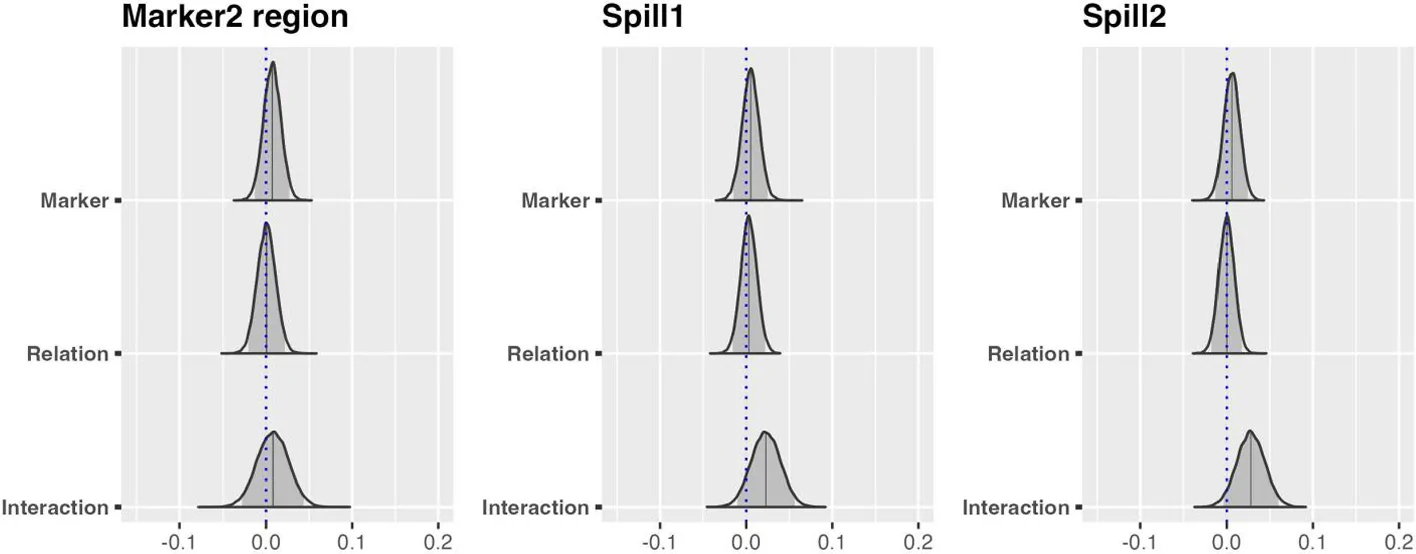
Posterior distributions for the comparisons between conditions, Exp. 2. The darker vertical line shows the median posterior effect estimate; the light gray area indicates the 95% credible interval

Figure 4 presents the reading times starting from Clause 1 through the Marker 2 region and to the subsequent spillover regions across conditions. Note that, as in Experiment 1, we see that Clause 1 appears to be read faster when Marker 1 is present compared to when it is absent. We return to this in the General Discussion. Posterior distributions of the estimated parameters are shown in Fig. 5, and full model results and a raw reading time plot are included in the Appendix (Table 8 and Fig. 8). Model results indicated no meaningful effects: all main effects and interactions were centered around zero. Note that the interaction on Spill2 almost excluded 0 in the 95% confidence interval.

**Fig. 6:**
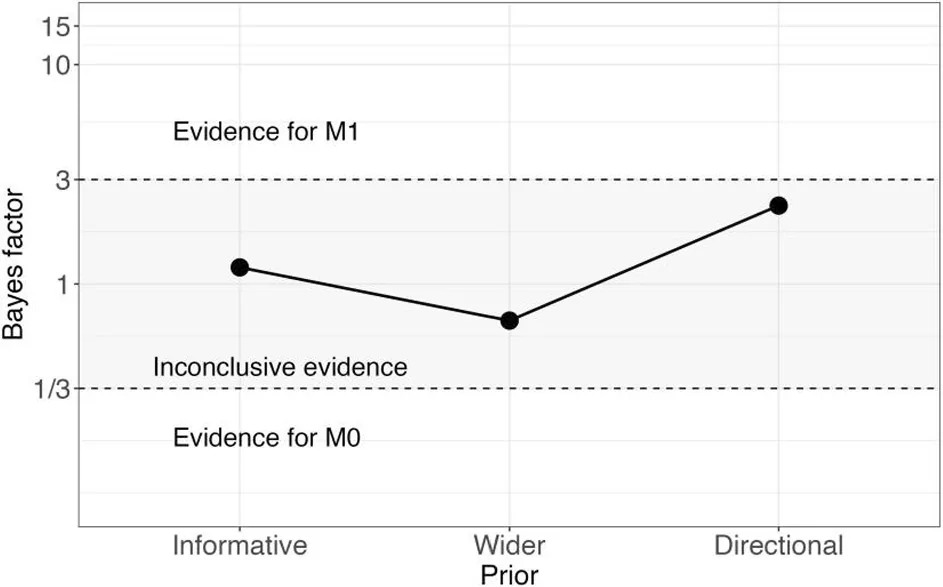
Bayes factor results for Model 1 over Model 0 for the interaction effect at the Spill2 region, Exp. 2

**Fig. 7:**
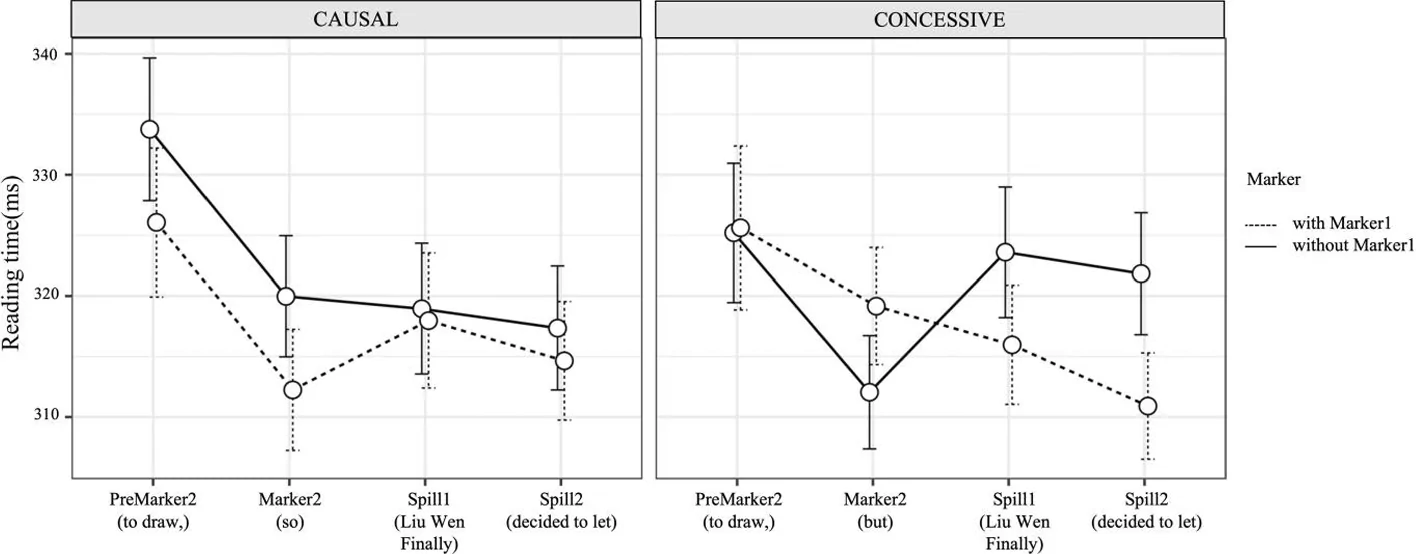
Mean reading times and error bars (SE) per region and condition, Exp. 1

**Fig. 8:**
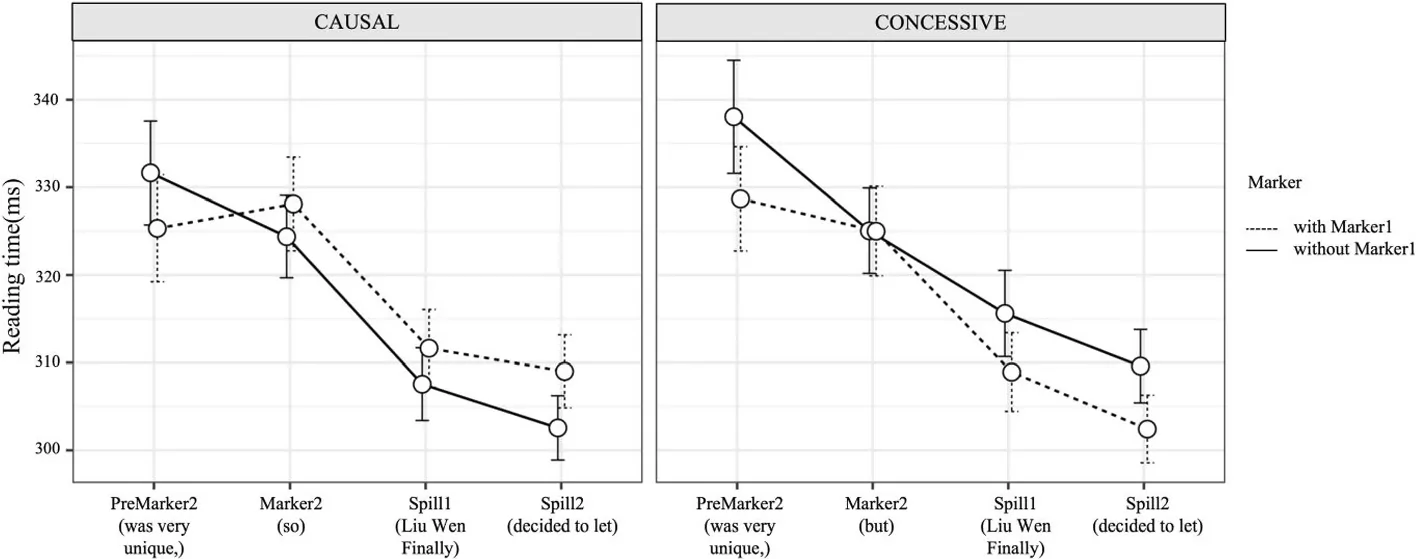
Mean reading times and error bars (SE) per region and condition, Exp. 2

*Bayes factor.* To quantify the strength of evidence for the interaction effect at the Spill2 region, we conducted Bayes factor analyses comparing models that included the interaction.

term (Model 1) to those that did not (Model 0). Figure 6 shows Bayes factors (BF_10_) for three prior specifications. Results indicated anecdotal to moderate evidence against an interaction effect.

*Exploratory: Pooled analysis.* To increase statistical power and assess the robustness of the observed interaction pattern across experiments, we conducted an exploratory combined analysis pooling data from both studies. This approach allowed us to examine whether the weak and partially inconsistent effects observed in the individual experiments reflect genuine but small processing differences or study-specific variability. The combined model included data from both experiments with Experiment entered as a between-subjects fixed effect. At the Marker 2 region, the results showed no interaction effect ($\hat{\beta }$ = −0.02, 95% CRI = (−0.05, 0.02)). At the spillover 2 region, the results showed a small interaction effect between marker and relation ($\hat{\beta }$ = 0.03, 95% CRI = (−0.00, 0.06)), similar in size and direction to the effect reported in Experiment 1.

### Discussion Experiment 2

This follow-up study was designed to test whether there is evidence that readers predict the lexical Marker 1 to Marker 2 in causal and concessive relations in Chinese, in an experimental design with more power and simpler items. Contrary to our predictions, there was no evidence that the presence of Marker 1 facilitated processing at Marker 2. Likewise, we found no reliable effect of relation type, suggesting that concessive relations were not more difficult to process than causal relations. Further, Experiment 1 suggested a possible interaction at the Marker 2 region in the opposite direction from our hypotheses, but this pattern did not replicate in Experiment 2, nor in the pooled analysis, which suggests that the effect should not be taken as strong evidence of a reliable processing pattern. Taken together, two experiments therefore converge on a null result: the presence of Marker 1 did not facilitate the processing of Marker 2, nor did relation type modulate processing.

It is possible that, even with simplified materials, the associative strength between the two markers in each pair was not sufficiently strong to produce facilitation effects. This interpretation is consistent with corpus evidence suggesting that two-part discourse markers in Chinese are relatively infrequent and loosely coupled, especially compared to more rigid connective systems in languages like English.

## Discussion

The present study investigated whether the processing advantage of two-part connectives observed in English extends to Chinese, and whether such facilitation is modulated by discourse relation type. Specifically, we asked whether the presence of the first marker facilitates processing of the second marker, and whether this facilitation is stronger for concessive than for causal relations. Based on prior evidence (Scholman et al., 2017, 2024, 2025; Schwab & Liu, 2020) and the complexity of concessive relations (Köhne-Fuetterer et al., 2021; Xu et al., 2018), we predicted (i) a main effect of relational marking, (ii) a main effect of relation type, and (iii) an interaction, with greater facilitation for concessive relations.

Across two self-paced reading studies, however, we did not find evidence for these predictions. In neither experiment did the presence of the first marker reliably facilitate reading times on the second marker or its subsequent region. Moreover, the predicted larger effect for concessive connectives relative to causal connectives was not reliable (Exp. 2) or inconsistent with predictions (Exp. 1). Although Experiment 2 showed a numerical interaction in the spillover regions, this pattern was not statistically reliable and did not replicate across experiments. However, the pattern was not statistically reliable: the Bayes factors indicated anecdotal to moderate evidence against an interaction effect. This is unlikely to reflect insufficient statistical power since Bayesian analyses allow us to distinguish between true null effects and inconclusive data. Moreover, the pattern did not replicate across experiments: Experiment 1 showed a different and inconsistent configuration. Together, these results do not support a stable facilitative effect of Marker 1 in Chinese.

Our prediction of stronger facilitation for concessive relations was motivated by two intertwined considerations: the higher cognitive demands of concessive relations and the stronger distributional coupling between concessive Marker 1 and Marker 2 observed in the corpus. Because these factors co-vary in our materials, the present design does not allow us to isolate whether potential facilitation would arise from relational complexity, lexical association strength, or their interaction. The absence of an interaction in the experiments therefore constrains both accounts jointly, but future studies manipulating different connective pairs within the same relation type will be needed to disentangle these sources of facilitation.

The present findings can be further understood by distinguishing two potential levels of facilitation. At a lexical level, Marker 1 may pre-activate a specific Marker 2 form, leading to faster processing when that form is encountered. At a discourse-integration level, facilitation would be reflected in easier construction of the coherence relation in downstream regions. However, we observed neither robust facilitation on Marker 2 (lexical level) nor reliable downstream advantages (integration level). This suggests that, in Mandarin Chinese, twopart connectives may function less as predictive cues and more as confirmatory devices.

An additional pattern emerging from the descriptive analyses is that regions within Clause 1 were read faster when Marker 1 was present than when it was absent. While this effect was not part of our a priori hypotheses and should be interpreted cautiously (particularly because Clause 1 consisted of multiple segments that were aggregated for visualization), it may suggest that Marker 1 provides an early structural or relational cue that facilitates local processing. Importantly, however, this early facilitation does not extend to the processing of Marker 2 or to downstream integration regions. Although Marker 1 can signal the type of discourse relation (e.g., causal or concessive), it does not uniquely determine whether a second marker will appear or which specific form it will take. The critical point at which the relation is explicitly confirmed and integrated into the discourse representation therefore remains Marker 2. The absence of facilitation at this stage indicates that Marker 1 does not yield robust expectation-based benefits for the processing of the connective or the relation itself.

Our findings are consistent with previous work on Chinese discourse processing. Although Xu et al. (2018) did find a processing difference between causal and concessive relations, they did not find clear early facilitation effects of either type of (one-part) connective. While Gao et al. (2024) did find a facilitative effect of the presence of a connective, they reported no facilitative effect of a full causal connective pair (Marker 1—Marker 2) compared to a single connective (Marker 2 alone). Our findings extend this work by examining not only causal relations but also concessive relations, which differ in cognitive complexity.

Thus, the findings in the current study should not be interpreted as a failure to replicate previous results from other languages, but rather motivate the possibility of cross-linguistic variation in discourse processing mechanisms. However, because the present study was not designed as a direct cross-linguistic comparison, differences with prior literature on two-part markers in English, German, and Dutch may also reflect methodological factors such as material structure, task demands, expectation adaptation, and statistical power. For example, self-paced reading measures provide coarse-grained evidence of processing difficulty and may not capture subtle differences in expectancy or integration. Previous studies reporting facilitation from two-part connectives in English and Dutch often (also) used eye-tracking or combined online measures with continuation tasks (Scholman et al., 2017, 2024), which may be more sensitive to fine-grained prediction effects. However, Xu et al. (2018) and Gao et al. (2024) also used eye-tracking to study discourse processing in Chinese, and found only limited effects of connectives; results from both studies indicated that connectives did not affect early processing measures as they do in other languages.

Thus, the convergence of null effects across the experiments reported in this paper as well as other Chinese studies (using different measures, materials, and participant samples) strengthens the reliability of the findings reported in this paper and motivates the hypothesis that the facilitative role of Marker 1 may be weaker or less systematic in Mandarin than in English, German and Dutch. This pattern may reflect, among other factors, differences in the linguistic inventory or in processing strategies.

A key structural difference between Chinese and English lies in the inventory and predictability of two-part connectives. In English, constructions like *on the one hand…on the other hand* have highly specific pairings with limited alternatives. In Chinese, by contrast, the first marker of a two-part construction may be compatible with multiple second markers, reducing its predictive specificity. For instance, *yinwei *because’ can pair with *suoyi*, *yinci*, or *yin’er* (all meaning’so/therefore’), each with slightly different discourse functions (see Sect. "Discourse processing and discourse relations"). Similarly, *suiran ‘*although’ can combine with connectives such as *danshi*, *keshi*, or *buguo* (all meaning’but’) with varying degrees of concessive force. This many-to-many mapping between first and second markers may reduce the predictive power of Marker 1, as it does not uniquely specify which Marker 2 will follow or even guarantee that an overt Marker 2 will appear at all. As a result, the cost of generating and maintaining predictions may outweigh potential benefits.

An alternative explanation relates to fundamental differences in processing mechanisms across languages. The facilitative effect observed in English, German, and Dutch may rely primarily on expectation-driven processing, whereby a discourse marker directly pre-activates a specific discourse relation or structural frame (Köhne-Fuetterer et al., 2021; Scholman et al., 2017; Xiang & Kuperberg, 2015), facilitating integration when the predicted second marker appears. Chinese discourse processing, by contrast, may be more integration-driven, such that connectives are used to confirm or clarify relations after semantic content and pragmatic context have already suggested a plausible link. Under such an account, the first marker does not strongly influence processing because readers do not rely heavily on form-based cues to generate discourse expectations. Instead, they primarily use semantic and pragmatic information to infer relations, with explicit connectives serving a confirmatory rather than predictive role (cf. Xu et al., 2018). Our results therefore motivate the hypothesis that the role of connectives in discourse processing may be subject to cross-linguistic variation (pending confirmation in directly comparable cross-linguistic studies).

One possibility is that this pattern relates to broader typological properties of Mandarin Chinese. Cross-linguistic corpus studies indicate that implicit discourse relations are more frequent in Chinese, with coherence relations often conveyed through semantic content, word order, and pragmatic inference rather than explicit connective marking (Kong et al., 2019; Zhou & Xue, 2012). Native Chinese readers are thus experienced with inferring discourse relations from context alone. Following Köhne-Fuetterer et al. (2021), the preceding context in the present experiments already established clear semantic alternatives (e.g., contrasting options or opinions). This may have enabled Chinese readers (more strongly than the English and German participants in Köhne-Fuetterer et al., 2021) to anticipate the upcoming discourse relation independent of any explicit marking, thereby weakening the predictive effect of the two-part structure.

These findings have implications for theories of prediction in language processing. Predictive processing frameworks (Kuperberg & Jaeger, 2016; Levy, 2008) have been successful in accounting for processing patterns in Indo-European languages. However, the current study aligns with recent proposals that prediction is not a universal, obligatory mechanism, but rather a strategic process that is modulated by language-specific properties (Huettig, 2015). The absence of robust form-based prediction effects in Chinese does not imply an absence of prediction altogether. Rather, Chinese readers may engage in prediction at the semantic and conceptual level (anticipating what will be said) while relying less on structural cues to predict how that structure will be marked. This suggests that cross-linguistic variation in prediction effects may reflect differences in where prediction is deployed (content vs. form) rather than whether prediction occurs.

Several specific follow-up studies would be valuable. First, a cross-linguistic eye-tracking study directly comparing Chinese and English processing of parallel materials could identify the precise time-course at which processing strategies diverge. Such a study could consider other two-part structures that show more tight connections in corpus. Second, investigating individual differences in predictive processing tendencies within Chinese speakers could reveal whether some comprehenders do show facilitation from Marker 1, which would suggest strategy variation rather than a categorical difference. Third, examining developmentally how Chinese children acquire sensitivity to two-part connectives could clarify whether integration-driven processing is learned or reflects fundamental constraints of the language. In conclusion, our study contributes to discourse processing research by providing a systematic investigation of two-part connective processing in Chinese. Our results suggest that facilitation effects at the second marker observed in English, German and Dutch may not generalize universally, thereby constraining and enriching our understanding of how different languages achieve discourse coherence. The current work highlights the importance of considering potential language-specific properties, such as connective inventory, mapping specificity, and pragmatic conventions, when developing theories of discourse processing.

## Data Availability

All materials, code and data are available on OSF:https://osf.io/frh6z.

## Appendix

See Figs. 7, 8 and Tables 7, 8.

**Table 7:** Coefficients from Bayesian mixed-effects model for the effect of marker, relation type and the interaction between them for each region, Exp. 1

	**Region**								
	*Marker 2*			*Spill1*			*Spill2*		
	Post. *β*^ˆ^	CI low	CI up	Post. *β*^ˆ^	CI low	CI up	Post. *β*^ˆ^	CI low	CI up
Intercept	5.71	5.65	5.76	5.72	5.66	5.77	5.71	5.67	5.76
Trial order	0.00	0.00	0.00	0.00	0.00	0.00	0.00	0.00	0.00
Marker	0.00	–0.02	0.02	0.01	–0.01	0.03	0.02	0.00	0.04
Relation	0.00	–0.03	0.02	0.01	–0.01	0.03	0.00	–0.02	0.02
Marker:Relation	0.04	–0.08	–0.01	0.02	–0.02	0.06	0.02	–0.02	0.05

**Table 8:** Coefficients from Bayesian mixed-effects model for the effect of marker, relation type and the interaction between them for each region, Exp. 2

	Region								
	*Marker 2*			*Spill1*			*Spill2*		
	Post. $\hat{\beta }$	CI low	CI up	Post.$\hat{\beta }$	CI low	CI up	Post.$\hat{\beta }$	CI low	CI up
Intercept	5.72	5.66	5.77	5.68	5.63	5.73	5.67	5.63	5.72
Trial order	0.00	–0.01	0.00	0.00	0.00	0.00	0.00	0.00	0.00
Marker	0.01	–0.01	0.03	0.01	–0.01	0.02	0.01	–0.01	0.02
Relation	0.00	–0.02	0.02	0.00	–0.02	0.02	0.00	–0.02	0.02
Marker:Relation	0.01	–0.03	0.04	0.02	–0.01	0.06	0.03	–0.00	0.06

## Footnotes

1 In a causal relation, one clause presents the cause of an event whose outcome is described in the other.In a concessive relation, a causal expectation is set up and then explicitly denied. See Section 2 for furtherdetails.

2 https://corpus.bfsu.edu.cn/info/1082/1782.htm
